# Contagious Health Risk and Precautionary Social Distancing

**DOI:** 10.3389/fpsyg.2021.685134

**Published:** 2021-06-14

**Authors:** Helge Giese, Martina Gamp, F. Marijn Stok, Wolfgang Gaissmaier, Harald T. Schupp, Britta Renner

**Affiliations:** ^1^Department of Psychology, University of Konstanz, Konstanz, Germany; ^2^Department of Interdisciplinary Social Science, Utrecht University, Utrecht, Netherlands

**Keywords:** risk perception, social network, contagious disease, flu, behavioral immune system

## Abstract

**Background:**

Since humans are social animals, social relations are incredibly important. However, in cases of contagious diseases such as the flu, social contacts also pose a health risk. According to prominent health behavior change theories, perceiving a risk for one’s health motivates precautionary behaviors. The “behavioral immune system” approach suggests that social distancing might be triggered as a precautionary, evolutionarily learned behavior to prevent transmitting contagious diseases through social contact. This study examines the link between personal risk perception for an infectious disease and precautionary behavior for disease-prevention in the context of social relationships.

**Methods:**

At 2-week intervals during the first semester, 100 Psychology freshmen indicated their flu risk perception, whether they had been ill during the previous week, and their friendships within their freshmen network for eight time points.

**Results:**

Social network analysis revealed that participants who reported a high flu risk perception listed fewer friends (*B* = −0.10, OR = 0.91, *p* = 0.026), and were more likely to be ill at the next measuring point (*B* = 0.26, OR = 1.30, *p* = 0.005). Incoming friendship nominations increased the likelihood of illness (*B* = 0.14, OR = 1.15, *p* = 0.008), while the reduced number of friendship nominations only marginally decreased this likelihood (*B* = −0.07, OR = 0.93, *p* = 0.052).

**Conclusion:**

In accordance with the concept of a “behavioral immune system,” participants with high flu risk perception displayed a social precautionary distancing even when in an environment, in which the behavior was ineffective to prevent an illness.

## Introduction

In the context of health and illness, social relationships are generally regarded as protective factors with positive effects on morbidity and even mortality (Uchino et al., 1996; Eng et al., 2002; Falagas et al., 2007; Nausheen et al., 2009; Hawkley and Cacioppo, 2010; Pinquart and Duberstein, 2010; Adams et al., 2015). For example, a meta-analytical review determining the extent to which social relationships influence the risk of mortality revealed a 50% increase in the survival likelihood of individuals with close social relationships, an effect size comparable to some more classic protective factors (Holt-Lunstad et al., 2010). However, in the context of contagious or infectious diseases such as HIV, tuberculosis, influenza, or the common cold, social contact also poses a risk to our health, since contagious pathogens can easily spread through social contact (Anderson and May, 1992). For example, people who are simply more sociable, and are therefore likely to come into contact with a greater number of people, are more susceptible to infectious disease transmission (Nettle, 2005; Murray and Schaller, 2016).

In contrast to other major diseases such as cardiovascular diseases, social contact may thus be perceived as a risk factor for contagious diseases such as the flu. Since perceiving a risk for one’s health motivates preventive behaviors (Leventhal, 1970; Rogers, 1975, 1983; Schwarzer, 1992, 2004, 2008; Witte, 1992; Sheeran et al., 2013), people might also be motivated to avoid pathogen carriers or potentially infectious social contacts in order to reduce their likelihood of being exposed to infectious pathogens (Mortensen et al., 2010; Barth et al., 2013; Sawada et al., 2018; Schmälzle et al., 2019). Supporting this notion, experimental studies have shown that people prefer to avoid those who they suspect to be carrying pathogens, as well as people who actually have a disease (Crandall and Moriarty, 1995; Park et al., 2003).

This reasoning accords with the concept of the “behavioral immune system” (Schaller et al., 2010; Neuberg et al., 2011; Schaller and Park, 2011; Murray and Schaller, 2016), which proposes that we are evolutionarily trained to be sensitive to social cues of infectious pathogens in other people (Schaller et al., 2010; Schaller and Park, 2011). Compared to biological immune systems (i.e., immunological defenses), which are invaluable at detecting and fighting pathogens post-infection within the body, the behavioral immune system has namely the unique adaptive value to proactively defend against infectious pathogens in the immediate environment before they can enter the body, avoiding the need for an energetically expensive physiological immune response (Mortensen et al., 2010; Murray and Schaller, 2016). Still, distancing in everyday life has not yet been observed and models that characterize changes in social contacts over time (Ripley et al., 2015; Giese et al., 2020) can provide novel insights into how the behavioral immune system impacts people’s everyday social life. Furthermore, this method allows further to scrutinize how effective this strategy is at avoiding actual contraction in a non-pandemic setting.

The present study combines the assumption that personal risk perception motivates precautionary behaviors, as proposed by common health behavior theories, with the idea of the specifically learned preventive social distancing derived from the behavioral immune system conception to explore how personal risk perception for a contagious disease is linked to illness-prevention behavior within the context of social relationships. Social network analysis was used within a newly-evolving social network to examine precautionary social distancing in the context of the flu. The present study aims to investigate (1) whether personal flu risk perception is associated with changes in social distancing in a social network, and (2) how flu risk perception and the social network are associated with changes in a person’s illness status. In line with the notion of a behavioral immune system and health behavior theories, we would expect social distancing of people with higher risk perceptions, social distancing of ill individuals, and an increased likelihood of illness when being more socially exposed.

## Materials and Methods

### Participants and Procedure

On October 21st 2014, a total of 117 people attending an introductory psychology class at the University of Konstanz were invited to take part in a social network study, and were informed about the study procedure. Of these, 113 ultimately registered as psychology freshmen and were eligible for participation. Over the course of the next 2 weeks, 100 freshmen consented to participate and were included in the study. There was no information on the 13 individuals deciding not to take part.

The present data was collected as part of the Social Network Study (SozNet), a larger research project on the antecedents and consequences of network formation and consolidation in a freshmen sample. Only the measures used in the present study will be described here. There is a detailed data description of the same dataset concerning perceived and actual alcohol consumption (Giese et al., 2019), and exposure effects (Giese et al., 2020) while previous cohorts of psychology freshmen networks are also presented elsewhere (Hartung and Renner, 2013, 2014; Giese et al., 2017).

In the last week of October, the participants indicated their age (*M* = 21.06; SD = 5.42) and gender (79% female) in a baseline questionnaire. Starting in the first week of November, and continuing until the third week of February, they provided biweekly information about their flu risk perception, whether or not they had been ill in the previous week, and their friendship nominations within the network of participating freshmen (as a proxy of social distancing). Invitations to complete the next questionnaire were sent out via email on Mondays. Completion was possible until Friday of the same week. All 100 consenting participants filled in the baseline questionnaire. Over the course of the semester, 762 out of 800 possible entries were given in a total of 8 questionnaires (4.75% attrition, see also Table 1). Participants who missed one questionnaire often still took part in later assessment periods and were too few for systematic drop-out analyses. Ethical approval was granted by the University of Konstanz ethical review board.

**TABLE 1:** Descriptive statistics.

Time point	Participants	Risk perception		Illness	Friends nominated	Jaccard index
	*n*	*M*	*SD*	(in %)	*M*	
1	99	2.93	1.18	40.4	3.91	
2	97	3.03	1.19	28.9	4.33	0.664
3	99	2.93	1.19	33.3	4.43	0.706
4	94	2.78	1.18	19.1	4.62	0.697
5	93	2.59	1.17	30.1	4.83	0.769
6	95	2.55	1.07	35.8	4.87	0.712
7	96	2.48	1.13	29.2	5.17	0.743
8	89	2.09	1.14	32.6	4.72	0.757
Overall	100	2.67	1.19	31.2	4.61	

**Notes* The Jaccard index is an index of friendship nomination stability between the given and previous time points, and is calculated by dividing the number of maintained nominations by the sum of all created, lost and maintained friendships. Thus, the friendships in the network were relatively stable.*

### Measures

#### Personal Flu Risk Perception

To assess personal flu risk perception, the participants estimated their own absolute risk of coming down with flu in the course of the semester (“How likely is it that you will come down with flu in the course of the present semester?”) on a 5-point Likert scale ranging from 1 “not very likely” to 5 “very likely” (Perloff and Fetzer, 1986; Renner and Reuter, 2012; Gamp et al., 2018). This measure was assessed at each time point.

#### Self-Reported Illness Status

To measure illness, the participants were asked each time point to indicate the number of days they had been ill in the previous week (“In the past week, how many days were you ill?”). Response alternatives ranged from 0 to 7 days. A binary illness status measure was computed from this item as required by the analysis method (Ripley et al., 2015): participants were categorized as having been ill if they reported at least 1 day of illness. If no ill days were indicated, illness status was categorized as “not ill” (Hartung and Renner, 2014). This measure pertained to any kind of illness.

#### Social Distancing Behavior

Each time point, every participant was provided with a list of the names and photos of all the other participants who had agreed to take part in the study. By referring to the list, the participants identified all the people they were befriended with (which was defined as feeling comfortable with and emotionally close to somebody, and being able to talk about personal issues or ask for help). All indications that were logically impossible were recoded as missing (e.g., if someone was indicated as being both a friend and as someone they did not like). The number of outgoing nominations was considered an indicator of proactive social distancing, while being able to control for effects of incoming nominations, which could be considered social avoidance by others.

### Analysis Strategy

In accordance with the notion of the behavioral immune system, the study explored (1) whether personal flu risk perception and illness status predict changes in friendship nomination behavior, and (2) how flu risk perception and the social network predict changes in a person’s illness status. To achieve these aims, a longitudinal social network model (RSiena, version 1.1-290; Ripley et al., 2015) was applied. This model uses the rate of chances to change for each individual between two adjacent time points and the current network structure, flu risk perception, and illness status to predict how likely a person is to change their friendship nominations and illness status. By simulating each change in status and friendship network, this analysis model can simultaneously predict changes in friendships as well as changes in illness status given the current friendship network, illness status, and risk perceptions. All estimates not pertaining to rates can be interpreted similarly to logistic regression estimates. The missing values (1–11% per time point) can be easily dealt with by the model (Ripley et al., 2015), which assumes no change for any missing value. While the RSiena model does not provide good power estimates, both the obtained group sample size and the number of observations compare well to simulations and indicate sufficient power to detect typical network effects (Stadtfeld et al., 2018).

Three core effects predicting friendship nominations are particularly relevant to our research questions (see also Table 2). (1) Activity effects describe the change in likelihood that an individual (ego) will choose to nominate another friend, given the current risk perception or illness status. (2) Popularity effects describe how much a change of one unit in risk perception or illness status at that moment changes the likelihood that an individual will be nominated by another individual (alter). (3) The interactions of activity and popularity effect estimate how similarity in risk perception or illness status changes the likelihood of a friendship nomination.

**TABLE 2:** RSiena model predicting changes in friendships and illness status.

Prediction of changes in friendships					

Name	Estimate	*SE*	OR	*z*-value	*p*-value
**Structural effects**					
Rate of decisions (Period 1)	2.96	0.33			
Rate of decisions (Period 2)	2.39	0.25			
Rate of decisions (Period 3)	2.69	0.30			
Rate of decisions (Period 4)	1.79	0.21			
Rate of decisions (Period 5)	2.54	0.29			
Rate of decisions (Period 6)	2.27	0.24			
Rate of decisions (Period 7)	1.98	0.23			
Out-degree (Density)	–2.75	0.13	0.06	–21.19	≤ 0.001
Reciprocity	3.06	0.13	21.42	23.09	≤ 0.001
Transitive triplets	0.57	0.04	1.77	14.62	≤ 0.001
3-cycles	–0.25	0.08	0.78	–3.29	0.001
In-degree popularity	0.06	0.02	1.06	3.27	0.001
Out-degree popularity	–0.19	0.02	0.83	–9.52	≤ 0.001
Out-degree activity	0.02	0.01	1.02	3.18	0.001
**Research questions**					
Illness popularity	–0.08	0.20	0.92	–0.39	0.695
Illness activity	0.37	0.24	1.44	1.50	0.133
Illness popularity × activity	0.89	0.63	2.43	1.41	0.158
Flu risk perception popularity	0.02	0.03	1.02	0.67	0.502
Flu risk perception activity	–0.10	0.04	0.91	–2.22	0.026
Flu risk perception popularity × activity	–0.04	0.03	0.96	–1.50	0.134

**Prediction of changes in illness**					

**Name**	**Estimate**	***SE***	**OR**	***z*-value**	***p*-value**

**Structural effects**					
Rate of chances to change (Period 1)	4.16	4.39			
Rate of chances to change (Period 2)	1.37	0.49			
Rate of chances to change (Period 3)	1.68	0.65			
Rate of chances to change (Period 4)	1.93	1.00			
Rate of chances to change (Period 5)	1.52	0.51			
Rate of chances to change (Period 6)	2.13	0.87			
Rate of chances to change (Period 7)	1.18	0.36			
Illness	–1.23	0.24	0.29	–5.11	≤ 0.001
**Research questions**					
Proportion of ill friends	0.14	0.71	1.15	0.20	0.839
Flu risk perception	0.26	0.09	1.30	2.82	0.005
Out-degree	–0.07	0.04	0.93	–1.95	0.052
In-degree	0.14	0.05	1.15	2.63	0.008

**Out-degree refers to the effects of the number of friends a person nominated as a predictor, in-degree refers to the same role for the number of received friendship nominations. The variables reciprocity, transitive triplets, and 3-cycle all structurally control for increased likelihood to nominate a friend back (reciprocity), or to nominate friend’s friends (both 3-cycle and transitive triplets). For predicting friendship network dynamics, activity effects refer to how the named predictor increases the likelihood of nominating someone, while the popularity effects describe how the variable increases the likelihood of receiving a nomination. The interaction activity × popularity tests how similarity in the predictor changes the likelihood of nomination. The structural effects are basic effects recommended by the developers of the modeling approach (Ripley et al., 2015) and control how the mere structure of the network (or the behavior) will influence changes in friendships and illness status. As such, for instance, the illness status effects controls for autoregression (namely that the likelihood of an illness at the next measurement decreases, when currently ill).**

Three additional effects are important for predicting changes in illness-status: (1) The average number of friends who are currently ill is a measure of contagion among friends, while (2) flu risk perception effects test whether a higher risk perception increases the likelihood of becoming ill. Finally, (3) in-degree and out-degree effects describe an individual’s likelihood of becoming ill depending on the number of people in the network he or she has nominated (out-degree) or how often that individual was nominated by others (in-degree).

Along with these six effects that are relevant for investigating the two research questions, the longitudinal social network model also includes effects that describe the structure of the network. This allowed us to control for the effects of previous friendships on subsequent friendship developments and general friendship nomination dynamics (Ripley et al., 2015). Hence, while these structural effects are not directly related to the present research questions, they facilitate the interpretation of the six core effects. We also explored the stability of the results by testing for the time differences of all effects via χ^2^ difference tests across all time points. All significant time differences in the full model predicting both changes in the friendship network and illness status were modeled via dummy coding to check for pattern changes across time (Lospinoso et al., 2011).

## Results

### Descriptive and Model Statistics

Table 1 provides descriptive statistics for all eight time points. Overall, flu risk perceptions were at a medium level (*M* = 2.67; SD = 1.19). Moreover, on average most participants rated themselves as being healthy (69%), while about one third stated that they were ill (31%). The average number of people the participants nominated as friends tended to increase from the start to the end of the study (*M* = 4.61). The presented social network model converged well, according to the fit criteria (Max_convergence ratio_ = 0.1297, all |*t*_convergence_| ≤ 0.047; Ripley et al., 2015).

### Main Analyses

Table 2 shows both structural effects and effects of interest on the likelihood of friendship nominations and illness status change^1^. There are three effects of both flu risk perception and illness status that test their effects on the development of friendship nominations: activity effects test how much risk perception/illness status increases the likelihood of a person nominating another person, popularity effects test whether a person is more likely to be nominated by others given their risk perception/illness status, and the interaction of the two tests whether nominations are more likely among similar others.

Of these effects, only flu risk perception activity was found to have a significant relationship with friendship nomination (*B* = −0.10, OR = 0.91, *p* = 0.026): the likelihood of nominating someone as a friend decreased as one’s flu risk perception was increased. Conversely, friendship nominations were not significantly predicted by flu risk perception popularity (i.e., risk perception did not have an effect on receiving nominations; *B* = 0.02, OR = 1.02, *p* = 0.502), the similarity of participants’ risk perception (popularity x activity; *B* = −0.04, OR = 0.96, *p* = 0.134), or any illness status effects (all *p* ≥ 0.133).

For predicting illness status, tests that evaluate whether the likelihood of becoming ill is increased by (1) the average illness status of all nominated friends (contagion), (2) the initial risk perception in this period, and (3) the number of incoming and outgoing friendship nominations (in-degree/out-degree) are particularly interesting. Analysis of these effects revealed no evidence for a contagion effect among friends (*B* = 0.14, OR = 1.15, *p* = 0.839): the proportion of friends being ill at any one moment did not change the likelihood of getting ill. However, people with a higher risk perception were more likely to become ill (*B* = 0.26, OR = 1.30, *p* = 0.005). Outgoing nominations marginally decreased the risk of becoming ill (*B* = −0.07, OR = 0.93, *p* = 0.052), while incoming nominations increased the risk significantly (*B* = 0.14, OR = 1.15, *p* = 0.008). Thus, it was more likely for students to become ill if many people nominated them as friends.

### Control Analysis: Stability of Effects Across Time Points

The effects described above are averaged across all time periods. The following control analyses tested the temporal stability of the results for all periods. While there were some time differences in the overall effects [χ^2^(108) = 301.00; *p* ≤ 0.0001], after accounting for temporal differences in all structural effects and the effects of flu risk activity and popularity on friendship nominations, the remaining effects did not show any other significant differences [χ^2^(54) = 63.26; *p* = 0.1818]. Considering these significant temporal differences, the effects for predicting friendship nominations changed only slightly compared to the general results: People with a high risk perception were not significantly less active from T3 to T4 (*B* = −0.19, OR = 0.83, *p* = 0.199) and T6 to T7 (*B* = 0.15, OR = 1.16, *p* = 0.483), and less popular from T2 to T3 (*B* = −0.32, OR = 0.72, *p* = 0.025). Therefore, the effect of flu risk perception activity on friendship nominations was found in five out of seven periods. The pattern of results remained stable for the prediction of illness status. Thus, the effects could be generally observed in most time spans and were not specific to single periods.

## Discussion

The present study examined how personal risk perception for a contagious disease is linked to social precautionary distancing within a newly-evolving social network. Building on previous research, the data shows that a high personal flu risk perception was related to a reduction in outgoing friendship nominations (risk perception activity). Or, to put it differently, participants who recognized a health threat also showed a more pronounced social precautionary distancing, which supports the behavioral immune system conception as well as health behavior theories attributing an important role to risk perceptions. However, changes in illness status were predicted by both higher numbers of received friendship nominations and an elevated risk perception, indicating that this distancing did not successfully prevent illness.

The observed social distancing supports and extends previous research in the realm of the behavioral immune system contention by showing an increased likelihood of social distancing in a naturalistic setting across longer time frames. Thus, the present results go beyond observational (Schaller and Murray, 2008), experimental (Mortensen et al., 2010; Sawada et al., 2018), or neuropsychological study designs (Stark et al., 2007; Baumann and Mattingley, 2012) to a more direct and “real-world” social behavior, namely the nomination of friendships within a time-span of a semester. Interestingly, this social distancing was confined to subjective risk perceptions, since illness status yielded neither decreased popularity nor additional distancing. In fact, the social distancing of the behavioral immune system might even be underestimated, since friendship nominations represent a high threshold dependent variable for precautionary distancing behavior in reaction to a relatively low health threat.

Strikingly, the (intentionally) lowered number of outgoing friendship nominations was only marginally related to the individual illness status, while personal flu risk perception predicted actual individual contraction of a disease. This implies that the observed social precautionary distancing could in this case actually be considered insufficient to prevent the disease. It seems that–although the participants were able to recognize an imminent health threat–this did not translate into effective precautions to decrease the risk of infection. Conversely, incoming friendship nominations from others, which are less under one’s individual control, significantly increased the likelihood of becoming ill, and could therefore be seen as an actual risk factor. In this light, one might also consider social distancing also as an altruistic measure to prevent others from contracting an illness.

Overall, while social contacts are seen as a protective factor with regard to non-commutable diseases (Uchino et al., 1996; Hawkley and Cacioppo, 2010), they also increase exposure to contagious diseases (Anderson and May, 1992). Social distancing as observed in this study may therefore be regarded as a learned precaution against risks such as contracting flu, potentially serving an evolutionary function (Nettle, 2005; Murray and Schaller, 2016). However, today’s mortality is mainly caused by non-communicable compared to contagious diseases. Accordingly, it is unsurprising that meta-analyses list social contacts as a protective factor for mortality (Holt-Lunstad et al., 2010). Moreover, as social contacts are not fully controllable and may buffer other risk factors such as stress (Uchino et al., 1996), social distancing may not be a sufficient, generally applicable control strategy for minimizing general health risks. Specifically, it should not be considered a substitute for other preventive measures such as hand hygiene (World Health Organization [WHO], 2010; Reuter and Renner, 2011) or vaccinations (Osterholm et al., 2012; Renner and Reuter, 2012).

### Limitations and Future Research Directions

However, this study has some caveats that limit the interpretation of the data. First, as we explored a dataset of one group and a mild contagious disease, the result should be replicated in a subsequent study. The group of psychology freshmen is a rather specific, non-representative convenience sample and, as network analyses required a confined group, friendships outside of this group were not considered. Thus, future studies may further scrutinize whether the found patterns generalize to a wider population. Likewise, future studies may include additional control variables to rule out potential alternative explanations.

Second, by exploring a given dataset, the validity of the measures was suboptimal: The flu risk perception was assessed by a single item, which tapped mainly into the perceived susceptibility of contracting the illness. Most noteworthy, we did not directly assess flu and flu-like illnesses, but rather self-reports of any illness. While this may explain the lack of contagion in the study, both the effect of flu risk perception on illness likelihood as well as the fact that the applied model ignores chronic conditions by solely modeling dynamics indicate that the assessment of illness at least partially captures the dynamics of flu infections. Furthermore, having friends does not require direct physical contact. While physical proximity may be essential to maintain friendships particularly in an newly evolving group like the one assessed (Giese et al., 2020), a measure of physical contact would be a more direct way of assessing social distancing. Still, findings of social distancing even on this measure of psychological proximity may indicate–in line with the notion of a behavioral immune system–that distancing is deeply ingrained even in absence of direct benefit. Future studies, may therefore employ a multi-item scale for perceived risk assessment and use a more direct–potentially even observational–measure of both physical contact and flu contraction.

Finally, some of the results need to be reaffirmed and further consequences of distancing should be considered in the light of COVID-19. For instance, future studies may also evaluate to what an extend social distancing due to high personal COVID-19 risk perception may actually lead to social isolation and thereby to a threat to psychological well-being, irrespective of its effectiveness in preventing this contagious disease.

## Conclusion

Members of a social network showed precautionary social distancing in reaction to elevated risk perceptions, indicating a behavioral immune system in reaction to contagious diseases. However, as the members of this social network only had partial control over their social contacts, the social distancing did not appear to be an effective strategy for illness prevention in the given environment.

## Data Availability Statement

The raw data supporting the conclusions of this article will be made available by the authors, without undue reservation.

## Ethics Statement

The studies involving human participants were reviewed and approved by Ethikkomission der Universität Konstanz. The patients/participants provided their written informed consent to participate in this study.

## Author Contributions

BR, FS, and HG conceptualized and designed the study. HG and FS were responsible for the acquisition of data. HG analyzed the data. All authors substantially contributed to the interpretation of the data. MG and HG drafted the manuscript, while the rest of the authors substantively revised it. All authors approved of the submitted version of the article and agreed to be accountable for their contributions and to ensure that questions related to the accuracy or integrity of any part of the work, even ones, in which the author was not personally involved, are appropriately investigated, resolved, and the resolution documented in the literature.

## Conflict of Interest

WG has received honoraria for lecturing/consultation from Amgen, Bayer, Biogen, Celgene, Genzyme, Merck Serono, MSD, Mundipharma, Novartis Pharma, Roche, Sanofi, and Teva, as well as research support from Biogen. The remaining authors declare that the research was conducted in the absence of any commercial or financial relationships that could be construed as a potential conflict of interest.

## Footnotes

1 Structural effects describe the effects of the network and behavior, respectively. They can be interpreted as follows: The rate effects describe how often an individual gets a chance to change nominations or behavior within the given 2-week period. Concerning friendship nominations, individuals were rather more likely not to nominate than to nominate a friend (out-degree). Similarly, people were more likely to nominate others that were already nominating them (reciprocity). Moreover, students were more likely to nominate peers nominated by another friend of that student (transitive triplets), but less likely to nominate peers that nominated a person already nominating the student (3-cycle). Both effects in combination indicate a local hierarchy in the network. Additionally, for each nomination given, it became less likely to receive a nomination (out-degree popularity), but more likely to give another one (out-degree activity). For each nomination received, it became more likely to receive another nomination (in-degree popularity). Concerning the behavior, individuals were more likely to change their illness status than to remain unchanged (illness).

## References

[B1] Adams R. N. Winger J. G. Mosher C. E. (2015). A meta-analysis of the relationship between social constraints and distress in cancer patients. *J. Behav. Med.* 38 294–305. 10.1007/s10865-014-9601-6 25262383 PMC4355094

[B2] Anderson R. M. May R. M. (1992). *Infectious Diseases of Humans: Dynamics and Control*, 28th Edn. Oxford: Oxford University Press.

[B3] Barth A. Schmälzle R. Renner B. Schupp H. T. (2013). Neural correlates of risk perception: HIV vs. leukemia. *Front. Behav. Neurosci.* 7:166. 10.3389/fnbeh.2013.00166 24312031 PMC3832789

[B4] Baumann O. Mattingley J. B. (2012). Functional topography of primary emotion processing in the human cerebellum. *Neuroimage* 61 805–811. 10.1016/j.neuroimage.2012.03.044 22465459

[B5] Crandall C. S. Moriarty D. (1995). Physical illness stigma and social rejection. *Br. J. Soc. Psychol.* 34 67–83. 10.1111/j.2044-8309.1995.tb01049.x 7735733

[B6] Eng P. M. Rimm E. B. Fitzmaurice G. Kawachi I. (2002). Social ties and change in social ties in relation to subsequent total and cause-specific mortality and coronary heart disease incidence in men. *Am. J. Epidemiol.* 155 700–709. 10.1093/aje/155.8.700 11943687

[B7] Falagas M. E. Zarkadoulia E. A. Ioannidou E. N. Peppas G. Christodoulou C. Rafailidis P. I. (2007). The effect of psychosocial factors on breast cancer outcome: a systematic review. *Breast Cancer Res.* 9:R44. 10.1186/bcr1744 17640330 PMC2206717

[B8] Gamp M. Schupp H. T. Renner B. (2018). Risk perceptions after receiving multiple risk feedback. *Personal. Soc. Psychol. Bull.* 44 1350–1363. 10.1177/0146167218767877 29716423

[B9] Giese H. Stok F. M. Renner B. (2017). The role of friendship reciprocity in university freshmen’s alcohol consumption. *Appl. Psychol. Heal. Well Being* 9 228–241. 10.1111/aphw.12088 28547919

[B10] Giese H. Stok F. M. Renner B. (2019). Perceiving college peers’ alcohol consumption: temporal patterns and individual differences in overestimation. *Psychol. Health* 34 147–161. 10.1080/08870446.2018.1514118 30295081

[B11] Giese H. Stok F. M. Renner B. (2020). Early social exposure and later affiliation processes within an evolving social network. *Soc. Netw.* 62 80–84. 10.1016/j.socnet.2020.02.008

[B12] Hartung F.-M. Renner B. (2013). Perceived and actual social discrimination: the case of overweight and social inclusion. *Front. Psychol.* 4:147. 10.3389/fpsyg.2013.00147 23554597 PMC3612696

[B13] Hartung F.-M. Renner B. (2014). The need to belong and the relationship between loneliness and health. *Z. Gesundheitspsychol.* 22 1–8. 10.1026/0943-8149/a000129 28847212

[B14] Hawkley L. C. Cacioppo J. T. (2010). Loneliness matters: a theoretical and empirical review of consequences and mechanisms. *Ann. Behav. Med.* 40 218–227. 10.1007/s12160-010-9210-8 20652462 PMC3874845

[B15] Holt-Lunstad J. Smith T. B. Layton J. B. (2010). Social relationships and mortality risk: a meta-analytic review. *PLoS Med.* 7:e1000316. 10.1371/journal.pmed.1000316 20668659 PMC2910600

[B16] Leventhal H. (1970). Findings and theory in the study of fear communications. *Adv. Exp. Soc. Psychol.* 5 119–186. 10.1016/S0065-2601(08)60091-X

[B17] Lospinoso J. A. Schweinberger M. Snijders T. A. B. Ripley R. M. (2011). Assessing and accounting for time heterogeneity in stochastic actor oriented models. *Adv. Data Anal. Classif.* 5 147–176. 10.1007/s11634-010-0076-1 22003370 PMC3192541

[B18] Mortensen C. R. Becker D. V. Ackerman J. M. Neuberg S. L. Kenrick D. T. (2010). Infection breeds reticence: the effects of disease salience on self-perceptions of personality and behavioral avoidance tendencies. *Psychol. Sci.* 21 440–447. 10.1177/0956797610361706 20424082

[B19] Murray D. R. Schaller M. (2016). “The behavioral immune system,” in *Advances in Experimental Social Psychology*, eds Zanna M. Olson J. (Amsterdam: Elsevier Inc.), 75–129.

[B20] Nausheen B. Gidron Y. Peveler R. Moss-Morris R. (2009). Social support and cancer progression: a systematic review. *J. Psychosom. Res.* 67 403–415. 10.1016/j.jpsychores.2008.12.012 19837203

[B21] Nettle D. (2005). An evolutionary approach to the extraversion continuum. *Evol. Hum. Behav.* 26 363–373. 10.1016/j.evolhumbehav.2004.12.004

[B22] Neuberg S. L. Kenrick D. T. Schaller M. (2011). Human threat management systems: self-protection and disease avoidance. *Neurosci. Biobehav. Rev.* 35 1042–1051. 10.1016/j.neubiorev.2010.08.011 20833199 PMC3024471

[B23] Osterholm M. T. Kelley N. S. Sommer A. Belongia E. A. (2012). Efficacy and effectiveness of influenza vaccines: a systematic review and meta-analysis. *Lancet Infect. Dis.* 12 36–44. 10.1016/S1473-3099(11)70295-X 22032844

[B24] Park J. H. Faulkner J. Schaller M. (2003). Evolved disease-avoidance processes and contemporary anti-social behavior: prejudicial attitudes and avoidance of people with physical disabilities. *J. Nonverbal Behav.* 27 65–87. 10.1023/A:1023910408854

[B25] Perloff L. S. Fetzer B. K. (1986). Self-other judgments and perceived vulnerability to victimization. *J. Pers. Soc. Psychol.* 50 502–510. 10.1037//0022-3514.50.3.502

[B26] Pinquart M. Duberstein P. R. (2010). Associations of social networks with cancer mortality: a meta-analysis. *Crit. Rev. Oncol. Hematol.* 75 122–137. 10.1016/j.critrevonc.2009.06.003 19604706 PMC2910231

[B27] Renner B. Reuter T. (2012). Predicting vaccination using numerical and affective risk perceptions: the case of A/H1N1 influenza. *Vaccine* 30 7019–7026. 10.1016/j.vaccine.2012.09.064 23046542

[B28] Reuter T. Renner B. (2011). Who takes precautionary action in the face of the new H1N1 influenza? Prediction of who collects a free hand sanitizer using a health behavior model. *PLoS One* 6:e22130. 10.1371/journal.pone.0022130 21789224 PMC3137604

[B29] Ripley R. M. Snijders T. A. Boda Z. Vörös A. Preciado P. (2015). *Manual for RSIENA.* Ocford: Univ. Oxford Dep. Stat. Nuff. Coll.

[B30] Rogers R. (1975). A protection motivation theory of fear appeals and attitude change. *J. Psychol.* 91 93–114. 10.1080/00223980.1975.9915803 28136248

[B31] Rogers R. (1983). “Cognitive and physiological processes in fear appeals and attitude change: a revised theory of protection motivation,” in *Social Psychophysiology: A Sourcebook*, eds Cacioppo J. Petty R. (New York, NY: Guilford Press), 153–177.

[B32] Sawada N. Auger E. Lydon J. E. (2018). Activation of the behavioral immune system: putting the brakes on affiliation. *Personal. Soc. Psychol. Bull.* 44 224–237. 10.1177/0146167217736046 29020867

[B33] Schaller M. Miller G. E. Gervais W. M. Yager S. Chen E. (2010). Mere visual perception of other people’s disease symptoms facilitates a more aggressive immune response. *Psychol. Sci.* 21 649–652. 10.1177/0956797610368064 20483842

[B34] Schaller M. Murray D. R. (2008). Pathogens, personality, and culture: disease prevalence predicts worldwide variability in sociosexuality, extraversion, and openness to experience. *J. Pers. Soc. Psychol.* 95 212–221. 10.1037/0022-3514.95.1.212 18605861

[B35] Schaller M. Park J. H. (2011). The behavioral immune system (and why it matters). *Curr. Dir. Psychol. Sci.* 20 99–103. 10.1177/0963721411402596

[B36] Schmälzle R. Hartung F.-M. Barth A. Imhof M. A. Kenter A. Renner B. (2019). Visual cues that predict intuitive risk perception in the case of HIV. *PLoS One* 14:e0211770. 10.1371/journal.pone.0211770 30785898 PMC6382111

[B37] Schwarzer R. (1992). “Self-efficacy in the adoption and maintenance of health behaviors: theoretical approaches and a new model,” in *Self-Efficacy: Thought Control of Action*, ed. Schwarzer R. (Washington, DC: Hemisphere), 217–243.

[B38] Schwarzer R. (2004). “Stage models of health behavior change: advances and problems,” in *Research on the Transtheoretical Model: Where Are We Now, Where Are We Going?*, eds Keller S. Velicer F. (Lengerich: Pabst Science Publishers), 110–113.

[B39] Schwarzer R. (2008). Modeling health behavior change: how to predict and modify the adoption and maintenance of health behaviors. *Appl. Psychol.* 57 1–29. 10.1111/j.1464-0597.2007.00325.x

[B40] Sheeran P. Harris P. R. Epton T. (2013). Does heightening risk appraisals change people’s intentions and behavior? A meta-analysis of experimental studies. *Psychol. Bull.* 140 511–543. 10.1037/a0033065 23731175

[B41] Stadtfeld C. Snijders T. A. B. Steglich C. van Duijn M. (2018). Statistical power in longitudinal network studies. *Sociol. Methods Res.* 2018:004912411876911. 10.1177/0049124118769113

[B42] Stark R. Zimmermann M. Kagerer S. Schienle A. Walter B. Weygandt M. (2007). Hemodynamic brain correlates of disgust and fear ratings. *Neuroimage* 37 663–673. 10.1016/j.neuroimage.2007.05.005 17574869

[B43] Uchino B. N. Cacioppo J. T. Kiecolt-Glaser J. K. (1996). The relationship between social support and physiological processes: a review with emphasis on underlying mechanisms and implications for health. *Psychol. Bull.* 119 488–531. 10.1037/0033-2909.119.3.488 8668748

[B44] Witte K. (1992). Putting the fear back into fear appeals: the extended parallel process model. *Commun. Monogr.* 59 329–349. 10.1080/03637759209376276

[B45] World Health Organization [WHO] (2010). *What Can I Do To Protect Myself from Catching Pandemic (H1N1) 2009?.* Available online at: http://www.who.int/csr/disease/swineflu/frequently_asked_questions/what/en/ (accessed June 30, 2017).

